# Looking for Minor Phenolic Compounds in Extra Virgin Olive Oils Using Neutron and Raman Spectroscopies

**DOI:** 10.3390/antiox10050643

**Published:** 2021-04-22

**Authors:** Roberto Senesi, Carla Andreani, Piero Baglioni, Luis A. E. Batista de Carvalho, Silvia Licoccia, Maria P. M. Marques, Giulia Moretti, Annalisa Noce, Roberto Paolesse, Stewart F. Parker, Enrico Preziosi, Giovanni Romanelli, Annalisa Romani, Nicola Di Daniele

**Affiliations:** 1 NAST Centre, Physics Department, Università degli Studi di Roma “Tor Vergata”, Via della Ricerca, Scientifica 1, 00133 Rome, Italy; roberto.senesi@uniroma2.it (R.S.); carla.andreani@uniroma2.it (C.A.); enrico.preziosi@uniroma2.it (E.P.); 2 CNR-IPCF Sezione di Messina, Viale Ferdinando Stagno d’Alcontres 37, 98158 Messina, Italy; 3 CSGI and Chemistry Department, University of Florence, Via della Lastruccia 3, Sesto Fiorentino, 50019 Florence, Italy; piero.baglioni@unifi.it (P.B.); moretti@csgi.unifi.it (G.M.); 4 Molecular Physical-Chemistry R&D Unit, Department of Chemistry, University of Coimbra, 3004-535 Coimbra, Portugal; labc@ci.uc.pt; 5 NAST Centre, Chemical Science and Technologies Department, Università degli Studi di Roma “Tor Vergata”, Via della Ricerca Scientifica 1, 00133 Rome, Italy; licoccia@uniroma2.it (S.L.); roberto.paolesse@uniroma2.it (R.P.); 6 Department of Life Sciences, University of Coimbra, 3000-456 Coimbra, Portugal; 7 UOC of Internal Medicine-Center of Hypertension, Nephrology Unit, Department of Systems Medicine, Università degli Studi di Roma “Tor Vergata”, Via Montpellier 1, 00133 Rome, Italy; annalisa.noce@uniroma2.it (A.N.); didaniele@med.uniroma2.it (N.D.D.); 8 ISIS Facility, STFC Rutherford Appleton Laboratory, Chilton, Didcot, Oxfordshire OX11 0QX, UK; stewart.parker@stfc.ac.uk; 9 PHYTOLAB (Pharmaceutical, Cosmetic, Food Supplement, Technology and Analysis)-DiSIA, University of Florence, Via U. Schiff, 6, 50019 Sesto Fiorentino, Italy; annalisa.romani@unifi.it

**Keywords:** extra virgin olive oil, minor polar compounds, inelastic neutron scattering, Raman spectroscopy, UV-Vis spectroscopy

## Abstract

Extra virgin olive oil (EVOO) is defined as a functional food as it contains numerous phenolic components with well-recognized health-beneficial properties, such as high antioxidant and anti-inflammatory capacity. These characteristics depend on their structural/conformational behavior, which is largely determined by intra- and intermolecular H-bond interactions. While the vibrational dynamics of isolated compounds have been studied in a number of recent investigations, their signal in a real-life sample of EVOO is overwhelmed by the major constituent acids. Here, we provide a full characterization of the vibrational spectroscopic signal from commercially available EVOO samples using Inelastic Neutron Scattering (INS) and Raman spectroscopies. The spectra are dominated by CH_2_ vibrations, especially at about 750 cm^−1^ and 1300 cm^−1^. By comparison with the spectra from hydroxytyrosol and other minor phenolic compounds, we show that the best regions in which to look for the structure–activity information related to the minor polar compounds is at 675 and 1200 cm^−1^ for hydroxytyrosol, and around 450 cm^−1^ for all minor polar compounds used as reference, especially if a selectively deuterated sample is available. The regional origin of the EVOO samples investigated appears to be related to the different amount of phenolic esters versus acids as reflected by the relative intensities of the peaks at 1655 and 1747 cm^−1^.

## 1. Introduction

Dietary products such as vegetable oils are known to contain biologically relevant components with recognized health-beneficial properties. Their phenolic constituents (namely phenolic acids and esters, e.g., cinnamic acid derivatives) display a well-known antioxidant activity, which makes them very effective chemo-preventive agents against oxidative-related diseases such as inflammation, cancer and neurodegenerative disorders [1,2,3,4,5].

Extra virgin olive oil (EVOO) is the main source of lipids in a Mediterranean diet (MD) [6,7,8,9]. Apart from the major triacylglycerols and unsaturated fatty acids, it comprises tyrosol (p-hydroxyphenylethanol), hydroxytyrosol (3,4-dihydroxyphenylethanol), lignans (e.g., (1)-pinoresinol and (1)-1-acetoxy-pinoresinol), secoiridoids (e.g., oleuropein and oleuropein aglycones) and relatively high amounts of at least 30 other minor polar phenolic acids/esters. The amount of these “non-nutrients” or minor polar compounds (MPCs) in EVOO may vary, depending on several factors, such as olive cultivar, location, climate, degree of maturation, agronomic and technological aspects of production [10]. Thanks to the protective role of these phenolic components against carcinogenesis, a direct relationship has been found between olive oil consumption and the incidence of different types of cancers [11] such as leukaemia [12], breast [13,14], prostate [15], lung [16], colon [17,18], bladder [19], carcinomas, cardiovascular diseases, and chronic kidney disease [20]. Recently, several studies have pointed out the nutraceutical role of EVOO in chronic non-communicable diseases (CNCDs) such as metabolic syndrome [21,22]. It is generally accepted that the cardioprotective properties of the MD are partly related to EVOO consumption [23,24]. Moreover, beneficial biological activities of polyphenols recovered from olive oil by-products and leaves have been described [21,22]. MPCs seem to exert a preventive and adjuvant action against CNCDs [23,25,26,27,28,29]. The study and analysis of the clinical and preclinical evidence of the cardiovascular beneficial effects of each constituent has been presented in Ref. [30].

Because of the beneficial properties of EVOO phenolic constituents, intense research has been directed towards the characterization of their structure–activity relationships (SAR). Vibrational spectroscopy is a powerful technique with which to characterize phenolic structures as, for example, it can provide information on the formation and chain length of alkyl esters and the intermolecular hydrogen-bond interactions inducing the dimerization of esters [31]. Complementary information can be obtained by the combined use of Raman spectroscopy and Inelastic Neutron Scattering (INS). In particular, INS exhibits a unique sensitivity to hydrogen, thus being an excellent technique for tackling H-bonding properties, such as the out-of-plane (O-H⋯O) mode, involving almost exclusively the motion of a hydrogen atom relative to the undeformed molecule [32]. Furthermore, INS yields intense features in the low-wavenumber region of the spectrum, where vibrations associated with hydrogen-type interactions mostly occur, thus providing complementary information to optical methods, such as infrared or Raman [33]. However, while a number of isolated components have been characterized using these techniques, as discussed later, an investigation of SAR of the MPC in a bulk sample of EVOO has not yet been presented. This is mainly due to the dominant vibrational signal arising from the major components of saturated and unsaturated fats, such as oleic, linoleic, and palmitic acids. While the spectroscopic fingerprint of isolated MPCs is a valuable piece of information, their vibrational frequencies and molecular structure are expected to change when a given MPC is considered within a real EVOO sample where intermolecular interactions are expected to be different from those present in the isolated extract of a given MPC [32].

The aim of this work is to assess possible strategies for the application of vibrational techniques, such as INS and Raman spectroscopies, to investigate the spectroscopic fingerprints of MPCs in EVOO samples. However, as the intensity in both INS and Raman spectra is proportional to the amount of a given molecule in the sample under investigation, the signal from MPCs is expected to be too weak to allow a detailed analysis of the peak position and, consequently, of the SAR. Therefore, the characterization of the predominant signal from major components in EVOO is a fundamental step towards the investigation of the spectroscopic fingerprints from MPCs. In particular, especially in the case of INS, once a promising energy region is recognized, where the signal from MPCs overlaps with a weakly structured background from major components, selective deuteration of the sample can be attempted to suppress the signal from the major components. In fact, the ratio of the neutron scattering cross sections of hydrogen and deuterium is about a factor of 11 [33]. As a result, one can eventually obtain the SAR of MPCs within EVOO samples, rather than from a pure extract following chemical separation.

In the present work, several EVOOs from the regions of Toscana, Umbria [34], Puglia, Lazio, and Abruzzo (Italy) were analyzed using INS and Raman spectroscopies. To the best of the authors’ knowledge, this was the first study of this type on dietary products by neutron scattering spectroscopy. The olive oil samples were obtained from olives grown organically or biodynamically with an organic oil label within the Italian and international market.

## 2. Materials and Methods

### 2.1. Materials

A series of EVOO samples were obtained from products commercially available in Italy: Toscana-Abruzzo blend (EVOO1); Toscana monocultivar 2019 (EVOO2); Umbria 2019 (EVOO3); Toscana monocultivar 2018 (EVOO4); Puglia monocultivar 2019 (EVOO5); Abruzzo monocultivar 2019 (EVOO6); Lazio blend 2018 (EVOO7); and Toscana monocultivar from biodynamic cultivation 2018 (EVOO8). Moreover, two standard samples were used: hydroxytyrosol (STD1) was obtained from olive pomace after EVOO production through a separation process by membrane technology followed by a concentration step under reduced pressure at low temperature, and STD 2 that was a fraction obtained from extraction and concentration from EVOO1 [21,24,35,36,37,38]. The EVOO samples were fully characterized prior to the spectroscopic measurements, namely regarding their acidity (% oleic acid), peroxide (mEqO_2_/kg_oil_) and total polyphenol content (mg_tyrosol_/kg_oil_), performed by using CDR-Oxitester system (CDR srl., Florence, Italy) and specific reagent kits [21]. A quasi-quantitative analysis of MPCs was performed by HPLC-DAD-MS using five-point regression curves built with the specific available standard references as reported in [21,35,36]. Solvents and reagents were purchased from Sigma Aldrich (Milan, Italy), hydroxytyrosol, tyrosol and oleuropein were furnished by Extrasynthèse, (Genay, France), and water was purified by a Milli-Q Plus system from Millipore (Milford, MA, USA).

### 2.2. INS Spectroscopy

INS measurements were carried out at the ISIS Pulsed Neutron and Muon Source of the STFC Rutherford Appleton Laboratory (Didcot, UK), using the time-of-flight high-resolution broad-range spectrometer TOSCA [39,40,41,42,43]. The samples were placed into indium-sealed thin-wall flat aluminum cans, with a 4 × 4 cm^2^ surface perpendicular to the neutron beam. To reduce the impact of the Debye–Waller factor on the observed spectral intensity, the samples were cooled to temperatures below 20 K. Data were recorded in the energy range from 0 to 4000 cm^−1^, and converted into the conventional scattering law, S(Q,ω) vs. energy transfer (in cm^−1^), using the MANTID program (version 5.1) [44]. All INS data were corrected by subtraction of an empty-can background and were normalized relative to the band at 720 cm^−1^ (assigned to the ρ(CH_2_) vibrational mode).

### 2.3. Raman Spectroscopy

Raman experiments were performed at the CSGI laboratories of the Chemistry Department of the University of Florence, using a Renishaw inVia Qontor confocal microRaman system equipped with 785 nm (solid state type, IPS R-type NIR785, 100 mW, 1200 L/mm grating) and 532 nm (Nd:YAG solid state type, 50 mW, 1800 L/mm grating) lasers, a research-grade Leica DM2700 microscope with a LWD50x objective (NA 0.55, WD 8.0 mm), LWD 100× (NA 0.75, WD 4.7 mm) and 100× (NA 0.85, WD 0.27 mm) objectives, and using a front-illuminated charge-coupled device (CCD) camera as a detector (256 × 1024 pixels, working temperature −70 °C). The 785 nm near-infrared laser was used as the excitation radiation, with a laser power at the sample varying between 0.6 and 3 mW, since these samples are very sensitive to the laser power, which can easily induce phenol dimerization and degradation processes. Spectra were collected in the range from 150 to 3500 cm^−1^ using the extended-range mode, an acquisition time of 10 s, and a single scan per sample. Raw data were pre-processed using the Renishaw software WiRE (version 5.2), and the baseline was corrected and normalized using the band at 720 cm^−1^ (assigned to the ρ(CH_2_) vibrational mode) as reference. The spectra were compared using Origin Pro 9.0 (OriginLab, Northampton, MA, USA).

### 2.4. UV-Vis Absorbance

Hexane solutions of EVOO8 and sunflower oil were investigated in the range 200–400 nm using a UV-Vis Varian Cary 50 spectrophotometer, with a resolution of 2 nm. Mixtures of a maximum of 20 μL of oil in 50 mL of hexane were used to avoid signal saturation. Hexane was chosen as the most suitable solvent to avoid absorbance bands from the solvent for wavelengths below 300 nm.

## 3. Results and Discussion

Table 1 shows the results of the EVOO HPLC-DAD-MS analysis and the result of acidity, peroxide content and total polyphenols analyzed with the CDR-Oxitester instrument. Furthermore, two standard samples were characterized. STD1 comprising 994.08 mg/g of hydroxytyrosol, and STD2 fraction obtained from extraction and concentration of EVOO1 containing hydroxytyrosol (12.38 mg/g), tyrosol (8.29 mg/g), elenolic acid (76.32 mg/g), 10-hydroxy-oleocanthal (287.62 mg/g), oleocanthal (189.16 mg/g), oleuropein aglycone (95.44 mg/g), ligstroside aglycone (23.29 mg/g) and ligustaloside A + B (171.99 mg/g), for a total of 864.49 mg/g MPCs.

**Figure 1:**
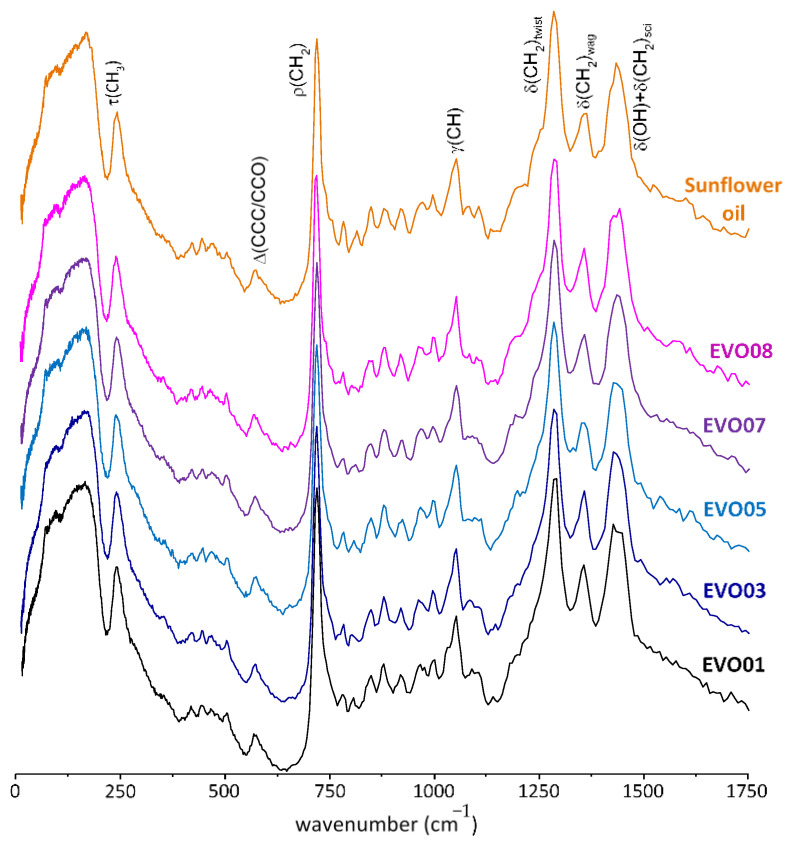
INS spectra (0–1750 cm^−1^) of some of the extra virgin olive oils (EVOOs) studied in the present work, and of sunflower oil.

**Figure 2:**
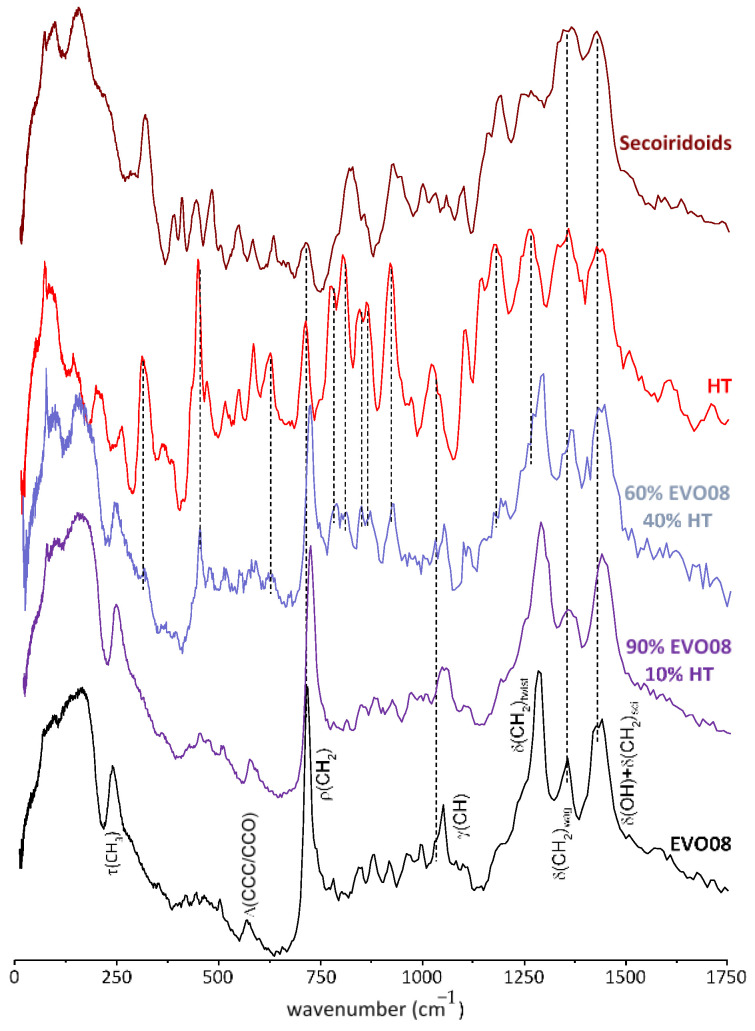
INS spectra (0–1750 cm^−1^) of extra virgin olive oil (EVOO8), hydroxytyrosol (HT) and secoiridoids, and two different mixtures of EVOO8 and HT (9:1 and 6:4)).

Figure 1 reports INS spectra obtained for some EVOO samples as well as sunflower oil. It is clearly seen that all the oil samples under analysis have very similar INS spectral signatures, which are dominated by the vibrational modes from unsaturated fatty acids, i.e., the major oil components: oleic (ca. 80%), linoleic and palmitic acids (ca. 20%) [45,46]. In addition, the profiles reveal the presence of hydroxytyrosol and secoiridoids (mainly oleuropein and oleuropein aglycone) (see Figure 2) that are important constituents of this vegetal oil (both from olives and olive leaves), [47] taken as reference compounds in this work. In particular, Figure 2 reports the spectra of 40% and 10% *w*/*w* STD1/EVOO8 mixtures. These spectra can be used as guides for the eyes to monitor the change in intensity of the characteristic peaks in pure hydroxytyrosol, present only as ca. 2 mg/L in EVOO8. It is interesting to note how in the region of the wagging δ(CH_2_) mode at ca. 1370 cm^−1^ and of the ρ(CH_2_) peak at ca. 720 cm^−1^, all spectra present well defined signals, which are the common signature of both MPCs and oil’s major components. On the other hand, hydroxytyrosol peaks between 1100 and 1200 cm^−1^ fall in a region where no intense structural features can be observed in EVOO8, and this is also the case for the peak at 675 cm^−1^, quite evident in hydroxytyrosol and in the 60%/40% EVOO8/STD1 mixture, corresponding to a smooth background signal in EVOO8. Moreover, a sharp peak at ca. 450 cm^−1^ in hydroxytyrosol corresponds to a region of EVOO samples with a weakly structured background in EVOO. To measure the SAR and the hydroxytyrosol’s dynamical effects in EVOO samples, the INS signals around 675 cm^−1^ and 1150 cm^−1^ are the most promising, as the spectral features from MPCs are isolated from the other bands ascribed to the major oil components (saturated and unsaturated fatty acids and esters).

**Figure 3:**
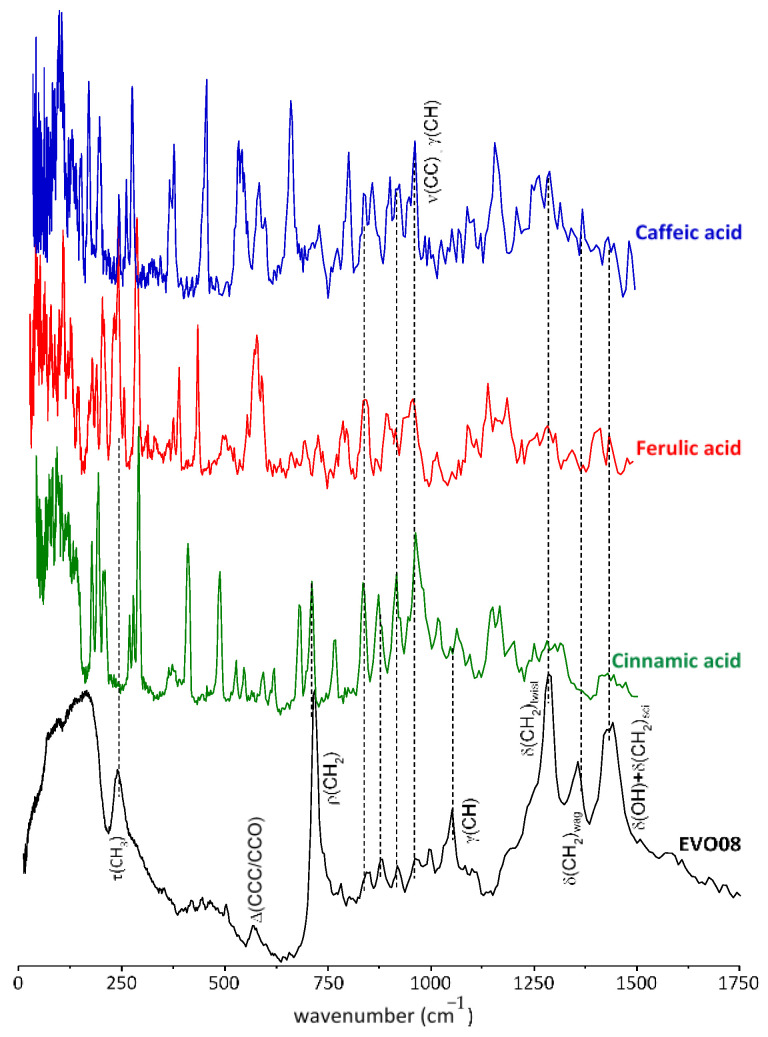
INS spectra (0–1750 cm^−1^) of extra virgin olive oil (EVOO8) and some representative dietary phenols (cinnamic, caffeic and ferulic acids).

EVOO phenolic constituents with recognized health-beneficial properties (e.g., coumaric, caffeic, ferulic and sinapic acids/esters), that were previously found to yield very definite vibrational signatures including INS characteristic bands [48] (Figure 3), are also MPCs in the present EVOO samples. However, their vibrational modes are largely obscured by those from the predominant non-phenolic elements. Figure 3 shows a comparison of the INS spectra from caffeic, ferulic, and cinnamic acids with that of EVOO8. In the case of caffeic acid, the most promising peak is the one at ca. 670 cm^−1^, where the signal from EVOO samples is relatively weak and unstructured, at energies slightly lower than the ρ(CH_2_) band. Moreover, for all MPCs in Figure 3, the region centered around 450 cm^−1^ is particularly interesting for the three reference molecules that present sharp peaks, possibly the γ(CH) band, although they fall in a weakly structured background in EVOO. Therefore, one can speculate that, by selectively deuterating the major components of EVOO, all four reference MPCs might be detected in this region.

**Figure 4:**
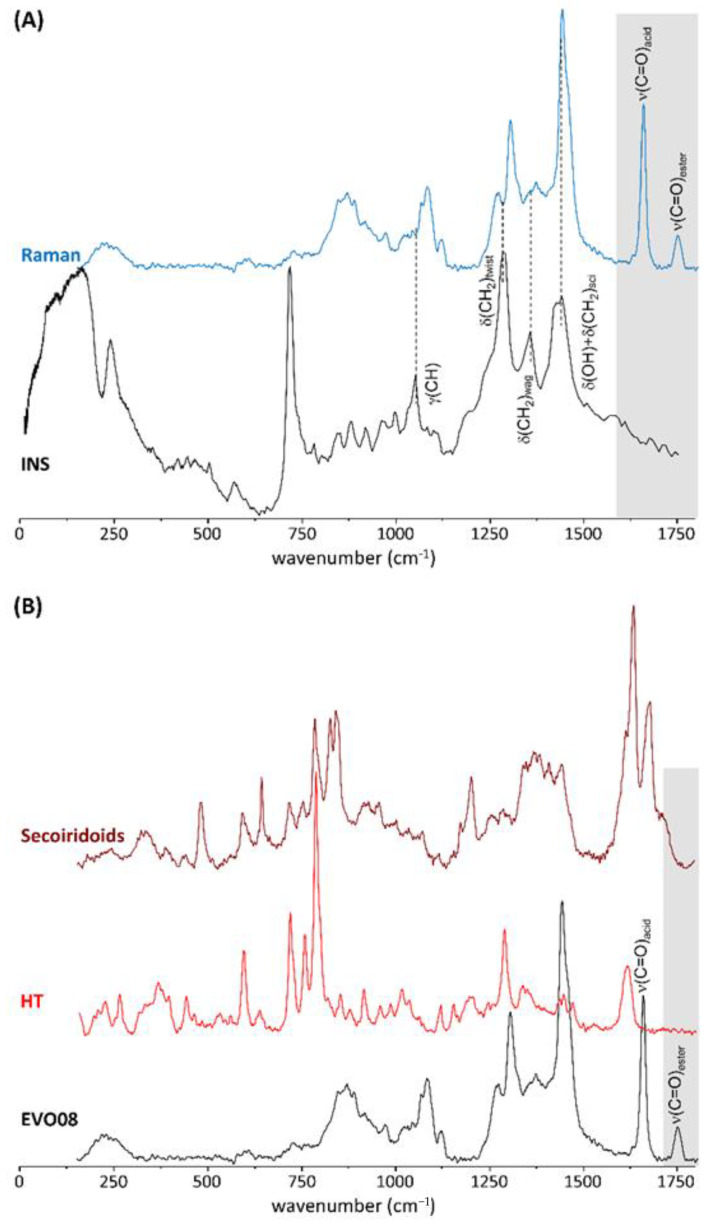
(**A**)—INS and Raman spectra (0–1750 cm^−1^) of extra virgin olive oil (EVOO8); (**B**)—Raman spectra (0–1750 cm^−1^) of EVOO8 and the reference compounds hydroxytyrosol and secoiridoids.

Figure 4 shows the Raman spectra recorded for the same samples. The spectra show some typical features of phenolic acids and esters [31,49,50,51]. In particular, the ν (C = O) mode detected at ca. 1747 cm^−1^, assigned to the esterified species, is not observed in the INS spectra as it does not involve hydrogen motion, as shown in Figure 4A. Also, the same peak is not present in either the secoiridoids or hydroxytyrosol constituents (Figure 4B). A detailed analysis of the Raman features of the carbonyl stretching modes characteristic of phenolic acids and esters, respectively at 1655 and 1747 cm^−1^, in the series of EVOOs and sunflower oil (Table 2), provides insight into the relative amount of phenolic esters versus acids in these samples. The I_1655_/I_1747_ ratio lies within the range from 4.39 to 4.59 for the olive oils, while a higher value of about 5 was obtained for sunflower oil, evidencing a higher content in esters for the EVOOs. The two monocultivar species from the Toscana region (Italy) (EVOO2 and EVOO4) display the lowest I_1655_/I_1747_ ratio (respectively 4.39 and 4.43), revealing a slightly larger amount of phenolic esters as compared to the other EVOOs.

The larger value of the I_1655_/I_1747_ ratio for sunflower oil, relative to other EVOO samples, is compatible with results reported in the literature [52], which revealed an intensity decrease in the peak at 1655 cm^−1^ for higher quality EVOOs with a lower unsaturation degree, while the peak at 1747 cm^−1^ was found to be relatively constant amongst samples. In our analysis, the significance of the changes in the ratio of the intensities at 1655 and 1747 cm^−1^ was checked against the ratios of peak intensities in the region 800–1200 cm^−1^. In the latter, that includes the CC stretching (869 cm^−1^) and bending (1079 cm^−1^) modes from the –(CH_2_)_n_– chains, the relative peak intensities were not found to vary significantly amongst the several samples, as expected [52].

**Figure 5:**
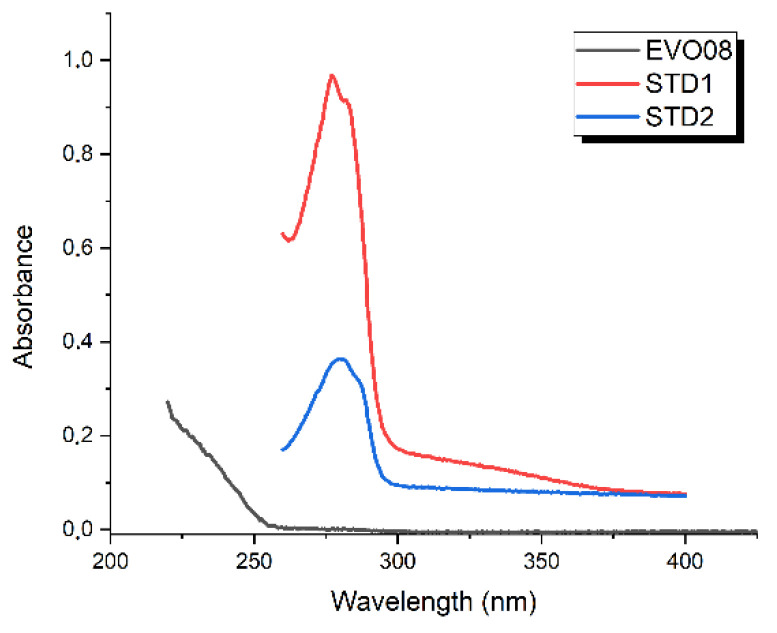
UV-Vis absorbance spectra of EVOO8 (black), STD1 (red) and STD2 (blue), in hexane solution (20 μL sample to 50 mL solvent).

Finally, Figure 5 shows the UV-Vis absorbance spectra of the EVOO8, STD1, and STD2 samples. The spectra of both STD1 and STD2 show a pronounced peak at about 280 nm, very similar to that found for oleuropein [10], making it difficult to discriminate among the several MPCs present in the EVOOs using this technique. Moreover, the 280 nm absorption becomes completely negligible in the case of the olive oil samples, as shown in Figure 5 for the particular case of EVOO8, which shows a sharp increase in the absorbance curve for wavenumbers lower than 255 nm.

## 4. Outlook and Conclusions

We have presented a spectroscopic investigation of a set of commercially available extra virgin olive oils (EVOOs) using Inelastic Neutron Scattering and Raman vibrational techniques. As the properties of different EVOO samples are related mainly to their minor phenolic components (MPCs), we find that different EVOO samples display similar INS and Raman signals. However, by comparison to the spectra from hydroxytyrosol and other MPCs, we show that the best regions to gain structure–activity information related to the MPCs are in the 675 and 1200 cm^−1^ range for hydroxytyrosol and around 450 cm^−1^ for caffeic, ferulic, and cinnamic acids. Our results provide a fundamental preliminary step in the investigation of the structure–activity relationship of minor polar compounds in real-life EVOO samples. From our results, future investigations based on INS, combined with selective partial deuteration to silence the vibrational contribution from the CH_2_ wagging modes of the major components in EVOO samples, or the structured signal around 450 cm^−1^, would open the way to a direct investigation of modes near these energies from the minor polar compounds. Finally, our results represent a step forward for accurate future investigations of the vibrational dynamics of olive oil samples subject to external stimuli, such as temperature or environmental parameters (e.g., soil composition and climate), as well as for the identification of minor polar compounds therein. For example, the detailed knowledge of the vibrational density of states, i.e., the signal measured by inelastic neutron scattering, could be used to analyze energy-resolved and/or time-resolved neutron imaging experiments [53] providing, e.g., molecular-specific information of the oil extraction process.

## Figures and Tables

**Table 1:** Minor polar compounds (MPCs) present in the extra virgin olive oils (EVOOs).

Constituents (mg/L)	EVOO1	EVOO2	EVOO3	EVOO4	EVOO7	EVOO8
hydroxytyrosol	3.12 ± 0.95	0.51 ± 0.02	0.98 ± 0.03	6.88 ± 0.28	1.47 ± 0.04	1.88 ± 0.06
Tyrosol	1.02 ± 0.04	1.23 ± 0.05	0.21 ± 0.01	6.22 ± 0.19	1.86 ± 0.06	1.87 ± 0.07
Elenolic acid derivatives	9.31 ± 0.28	60.8 ± 1.8	12.5 ± 0.4	36.6 ± 1.5	21.2 ± 0.64	29.3 ± 0.9
Elenolic acid	150 ± 4	31.5 ± 1.3	111 ± 3	106 ± 4	117 ± 3	197 ± 6
10-hydroxy-oleocanthal	315 ± 12	362 ± 11	61.0 ± 1.8	168 ± 7	67.5 ± 2.0	124 ± 4
Oleocanthal	198 ± 6	192 ± 6	46.2 ± 1.4	79.4 ± 3.2	94.1 ± 2.8	44.0 ± 1.3
Secoiridoid derivatives	96.4 ± 2.9	17.1 ± 0.7	30.9 ± 0.9	47.3 ± 1.9	48.4 ± 1.4	36.4 ± 1.1
Lignans	208 ± 8	160 ± 5	107 ± 3	90.2 ± 3.6	62.1 ± 1.9	129 ± 4
Oleuropein aglicone	164 ± 5	67.7 ± 2	105 ± 3	108 ± 4	83.4 ± 2.5	143 ± 4
**Total MPCs**	1146 ± 34	893 ± 27	475 ± 14	649 ± 19	497 ± 14	707 ± 21
Acidity (% oleic acid)	0.17 ± 0.01	0.15 ± 0.01	0.24 ± 0.01	0.16 ± 0.01	0.16 ± 0.01	0.15 ± 0.01
Peroxides (mEq O_2_/kg_oil_)	4.98 ± 0.20	5.21 ± 0.21	5.01 ± 0.15	8.82 ± 0.26	8.81 ± 0.26	7.81 ± 0.23
Polyphenols (mg_tyrosol_/kg_oil_)	890 ± 36	791 ± 31	354 ± 10	423 ± 13	342 ± 10	443 ± 13

**Table 2:** Raman intensity ratios for the bands at 1655 and 1747 cm^−1^ (νC = O from phenolic acids and esters, respectively), for the extra virgin olive oils and sunflower oil in the present study.

Sample	Source	I_1655_/I_1747_
EVOO1	Toscana–Abruzzo blend	4.52
EVOO2	Toscana monocultivar 2019	4.39
EVOO3	Umbria 2019	4.58
EVOO4	Toscana monocultivar 2018	4.43
EVOO5	Puglia monocultivar 2019	4.59
EVOO6	Abruzzo monocultivar 2019	4.50
EVOO7	Lazio blend 2018	4.55
EVOO8	Toscana monocultivar from biodynamic cultivation 2018	4.53
Sunflower oil		5.02

## Data Availability

The data presented in this study (TOSCA, RB2000118) are openly available at https://doi.org/10.5286/ISIS.E.RB2000118-1 (accessed on 21 April 2021).
